# Supply and demand in the physician workforce: an integrative review

**DOI:** 10.47626/1679-4435-2022-1022

**Published:** 2024-09-24

**Authors:** Monica Concha-Amin, Carolina Sturm Trindade, Paulo Ricardo Gazzola Zen, Bruna Bastos Giergowicz

**Affiliations:** 1 Department of Exact and Applied Social Sciences, Universidade Federal de Ciências da Saúde de Porto Alegre (UFCSPA), Porto Alegre, RS, Brazil; 2 Department of Clinical Medicine, UFCSPA, Porto Alegre, RS, Brazil; 3 Nursing School, UFCSPA, Porto Alegre, RS, Brazil

**Keywords:** labor, review, physicians, job market, medical economics, trabalho, revisão, médicos, mercado de trabalho, economia médica

## Abstract

The medical profession faces multiple challenges worldwide in the 21st century. This integrative review of the literature published between 2014 to 2018 on supply and demand in the health care labor market was based on a search of the SciELO, *Biblioteca Virtual em Saúde* (Virtual Health Library), and MEDLINE databases, in addition to specific periodicals in the area of Health Economics. The final sample included 21 studies, 13 of which were related to workforce supply and 8 to demand. Physicians reported that work satisfaction was an important factor in accepting a job offer, including being close to their family, working with colleagues in the same specialty, and the possibility of career advancement. For health system users, a fundamental question is how to resolve the unequal distribution of physicians.

## Abstract (pt)

A profissão médica se defronta globalmente com múltiplos desafios no século XXI, entre os quais podem-se destacar o crescimento populacional e a inovação tecnológica e a sua implementação na medicina. Este artigo buscou identificar na literatura científica aspectos relevantes acerca do mundo do trabalho para a profissão médica. Trata-se de uma revisão integrativa, que considerou as publicações do quinquênio de 2014 a 2018. A seleção dos artigos para o presente estudo foi realizada nas bases de dados Scientific Electronic Library Online, Biblioteca Virtual em Saúde, Medical Literature Analysis and Retrieval System Online, além de periódicos específicos da área de economia e saúde. Como resultado desta revisão, foram selecionados 21 artigos, sendo identificados 13 com temáticas referentes à oferta da força de trabalho médico e 8 com temáticas referentes à demanda da força de trabalho, de acordo com a classificação que emergiu pelos critérios adotados. Para os profissionais médicos, um aspecto que surge durante a decisão de escolher uma vaga é a satisfação pessoal no emprego, no sentido de estar perto da família, trabalhar com colegas da mesma especialidade e poder avançar na profissão. Já com relação à demanda da força de trabalho, ou seja, para quem busca os serviços de tais profissionais, uma questão fundamental é como resolver problemas de escassez de profissionais médicos que estão relacionados a uma distribuição desigual em determinados locais.

## INTRODUCTION

The study of markets is of vital importance in economics.^1^ The supply and demand of medical labor is an intrinsic determinant of health care system function, whether public, private, or mixed.

A market exists if there is supply, demand, and a specific good or service to be transacted.^1,2^ In the case of the physician workforce, physician labor is the transacted good or service, hence supply. The demand for labor from this workforce is a derived demand,^3^ since what the population seeks is health care, which can be provided directly through private practice or through health system intermediaries, such as health insurance, public or private hospital systems, philanthropic medical services, primary care, etc.

Job openings are being transacted in this market, which are a distinct asset from the workforce. Job openings are offered by the health system, whether in the public, private, or non-profit sector. If job openings are the source of work, then physicians offer their services to fill job openings that the market offers.

Thus, if the physician workforce refers to the labor of physicians, the demand comes from public or private sector health system users who seek services from the medical workforce. The terms “market”, “supply” and “demand” are used in this article conceptually. However, we are not suggesting a marketing vision for this profession or for medical care.

Determinants of physician workforce supply include the region’s level of social and economic development, the number of available medical schools, and the specific conditions of professional training and performance, the number of available physicians, remuneration, whether as wages or fees per procedure, the opportunity cost (measured in terms of alternative sources of revenue), and other expenses associated with professional practice, such as traveling between hospitals and leasing/purchasing office space.^2,3^

There are also intangible costs, such as the risk of contracting a disease from a patient and a work environment that involves precarious conditions, violence, professional devaluation, etc. The socioeconomic characteristics of physicians also influence their position and place of work, including sex, age, marital status, number of children, place of birth, alma mater, residence of family members, preferred residence area (urban or rural), the search for greater professional specialization, etc.^2,3^

Determinants of workforce demand include the number of health care institutions (public or private hospitals, private care clinics, health insurance operators, etc.) that need physicians, the number of job openings in the public health network, medical legislation, the population’s epidemiological situation, the demographic pyramid, the country’s socioeconomic characteristics, and the international mobility of labor. In this market, price is defined as wages or fees for medical procedures in different forms of remuneration.^2,3^

The present study reviewed scientific literature related to labor market issues for physicians, considering aspects of workforce supply and demand.

## METHODS

This integrative literature review^4^ was conducted in 6 steps: (1) formulating the research question, (2) establishing inclusion and exclusion criteria for studies or literature search parameters, (3) determining the types of data to be extracted from the studies and study categorization, (4) evaluating the included studies, (5) interpreting the results, and (6) presenting the review.

In stage 1, the following research question was determined: “What scientific knowledge has been published on labor market issues for physicians, especially regarding workforce supply and demand and remuneration models?”

In stage 2, the literature search was performed between August and September 2018 in the SciELO, *Biblioteca Virtual em Saúde* (Virtual Health Library), and MEDLINE databases. The following journals were also searched: *Health Economics, Health Economics Review, International Journal of Health Economics Management, Journal of Health Economics,* and *Nordic Journal of Health Economics.* These journals were suggested through a search of the Periodical Portal of the Coordination for the Improvement of Higher Education Personnel (an agency of the Brazilian Ministry of Education) using the term “Health and Economics”. The keywords and search expressions are presented in Table 1.

**Table 1:** Database search strategy

Search expression by database/journal	
SciELO	(Mercado de Trabalho OR Mercado de Trabalho Brasileiro OR Mercado de Trabalho dos Jovens OR Mercado de Trabalho Médico) AND (Medicina OR Médico)
MEDLINE	(((“Labor Market”) OR (“Job Market”))) AND ((“Physicians”[Mesh]) OR “Medicine”[Mesh])/Filtro
BVS (1)	(Mercado de Trabalho) AND (Medicina OR Médico)
BVS (2)	(Mercado de Trabalho) AND (Medicina OR Médico) AND (Juventude OR Jovem)
HE, HER, IJHEM, JHE, NJHE	Labor Market AND Physician

BVS = *Biblioteca Virtual em Saúde;* HE = *Health Economics;* HER = *Health Economics Review;* IJHEM = *International Journal of Health Economics and Management;* JHE = *Journal of Health Economics;* MEDLINE = Medical Literature Analysis and Retrieval System Online; NJHE = *Nordic Journal of Health Economics;* SciELO = Scientific Electronic Library Online.

The inclusion criteria were original full-text articles published in Portuguese, English, or Spanish between 2014 and 2018 that simultaneously addressed the medical profession and the job market. Duplicate articles, theses, dissertations, documents, letters to the editor, editorials, and books, in addition to studies that did not fit the review’s objectives were excluded. The articles were grouped into the following 4 thematic categories: (1) supply and demand for physicians, (2) remuneration models, (3) medical education, and (4) other. We decided to include only studies in categories 1 and 2, which better conformed with the study question.

The study selection was performed independently by 2 reviewers. A third reviewer was called in cases of disagreement. The initial selection was based on a reading of the titles and abstracts, identifying studies that might meet the inclusion criteria. Of a total of 310 identified records, 58 were considered eligible and were classified according the above mentioned thematic categories. A total of 48 articles passed this round of screening and were then read in full. Of these, 21 were considered eligible for the review, as shown in Table 2.

**Table 2:** Study selection process

Article search in databases and journals								
SciELO	MEDLINE	BVS (1)	BVS (2)	HE	HER	IJHEM	JHE	NJHE
23	62	52	21	20	16	26	90	0
310 results								
Title and abstract reading								
SciELO	MEDLINE	BVS (1)	BVS (2)	HE	HER	IJHEM	JHE	NJHE
7	17	17	2	7	0	1	7	0
58 eligible articles								
Initial reading and application of the classification criterion								
Classification		SciELO	MEDLINE	BVS (1)	BVS (2)	HE	IJHEM	JHE
1(17)		0	8	5	1	2	1	0
2(9)		0	5	1	0	1	0	2
3(14)		0	2	8	0	2	0	2
4(8)		0	1	2	0	2	0	3
Total per database/journal		0	16	16	1	7	1	7
Total of 48 articles: articles classified as category 3 or 4 were excluded										
Detailed reading (category 1 and 2 articles)								
MEDLINE				BVS (1)	BVS (2)	HE	IJHEM	JHE
9				6	1	2	1	2
21 articles included in the review								

BVS = Biblioteca Virtual em Saúde; HE = Health Economics; HER = Health Economics Review; IJHEM = International Journal of Health Economic and Management; JHE = Journal of Health Economics; MEDLINE = Medical Literature Analysis and Retrieval System Online; NJHE = Nordic Journal of Health Economics; SciELO = Scientific Electronic Library Online.

In step 3, data collection and synthesis, an instrument was created to collect the following data: article identification (author, year, and *Journal of Economic Literature* code when found in the original text); description, objectives, and study location; methodology; and main results, classified according to the following codes: I11 (Analysis of Health Care Markets), I12 (Health Behavior), I18 (Government Policy, Regulation and Public Health), J21 (Labor Force and Employment, Size, and Structure), J22 (Time Allocation and Labor Supply), J44 (Professional Labor Markets and Occupational Licensing), L11 (Production, Pricing, and Market Structure, and Size Distribution of Firms), L78 (Government Policy), and 05 (Economywide Country Studies), which are available at https://www.aeaweb.org/econlit/jelCodes.php?view=jel#J.

Ethical aspects were ensured in this integrative review, guaranteeing the authorship of the articles. The authenticity of the ideas, concepts and definitions of the authors included in this review were maintained. This study was registered with the Federal University of Health Sciences of Porto Alegre Research Committee.

To analyze a market, the good or service in question must be defined, which, in this case, is the physician workforce. We used the partial equilibrium model, developed by economist Alfred Marshall in the 19th century,^5^ which assesses one variable while keeping others that might influence it constant and, thus, in equilibrium. Hence, when analyzing the physician workforce as a market, the supply and demand of this good must be analyzed while keeping all other variables that could create changes simultaneously constant (*ceteris paribus*).

Steps 4, 5, and 6, previously described by Botelho et al.,^4^ are elucidated in the results, discussion, and conclusion.

## RESULTS

Tables 3 and 4 provide a summary of the data from each article regarding supply and demand in the physician workforce, respectively. Thirteen studies (61.9%) addressed supply (Table 3) and 8 (38.1%) addressed demand (Table 4).

**Table 3:** Data synthesis on physician workforce supply in the medical labor market

Article (author, year and JEL codes)	Description, objectives, and study location (SL)	Methodology	Main results
Leung et al.,^6^ 2018	Surveyed the views of recent oncology graduates about entering the workforce. SL: Australia and New Zealand	Questionnaire covering work conditions, relocation to rural or urban areas, current employment, job search experiences, job satisfaction, and perceptions about the job market.	The number of weeks spent searching for a position depend on the job type; high degree of job satisfaction (75%); only 27% worked in rural areas; most agreed that better workforce planning is needed.
Chen et al.,^7^ 2018	Determined how the 2009 expansion of a public pediatric health care insurance program affected pediatricians. SL: United States	Center for Health Workforce Studies database of physicians in residency, training, and initial employment in New York State, USA.	The new law led to important supply-side changes: pediatricians were more likely to specialize or enter private practice after, including growth in rural private practice.
Kalb et al.,^8^ 2018, J22; J44; J21	Determine the response of general practitioners and specialists to simulated wage changes. SL: Australia	Data from the 2008 Medicine in Australia: Balancing Employment and Life survey was used to estimate 3 utility specifications, including 1 separately for men and women, with the dependent variable being the number of hours worked.	Wage increases led to a slight reduction in supply. Only married female specialists with unemployed husbands slightly increased the number of hours worked.
L’hopital,^9^ 2017	Studied the socioeconomic characteristics, employment, and motivation in medical workforce supply. Describes the employment situation of graduates of the General Medicine Residency System in the province of Buenos Aires between 2008 and 2013. SL: Argentina	Survey of the labor status of general practitioners who completed their residency between 2008 and 2013.	The vast majority were women (79.4%) and worked in the public sector (79%), with many having 2 or 3 jobs (35% and 27.9%, respectively) The mains reasons cited for working in the public sector were the teamwork and additional benefits.
Suciu et al.,^10^ 2017	Assessed the intent of Romanian medical school graduates to emigrate within the EU due to increased relaxation of labor restrictions. SL: Romania	A 19-item questionnaire was applied each year from 2013 to 2015 at a medical university in Romania.	Of 957 respondents, 84.8% plan to seek employment abroad after graduation. The mean probability of emigrating was 42.17%, with no significant sex differences. The most common destinations were Germany (34.1%) and France (20%).
Maceira et al.,^11^ 2017	Determined the contractual characteristics of physician wages in a decentralized public health system. SL: Argentina	Multiple-choice questionnaires were applied to a representative sample of physicians from 5 jurisdictions (n=615). Secondary data on wages from September to October 2013 were obtained from each Ministry of Health.	Most physicians (75%) were under permanent state contracts. A diversity of contractual and structural arrangements and salaries were found between jurisdictions and among hospitals in the same jurisdiction. There was a high rate of contractual formalization.
Higgins et al.,^12^ 2016	Assessed workforce demographics among faculty and fellows in interventional radiology. SL: United States	Statistical comparison of demographic representation based on U.S. Census data.	Women, Blacks, and Hispanics were underrepresented. No significant increase in female or Black representation for the last 20 years.
Arruda et al.,^13^ 2016	Analyzed job availability in plastic surgery and the profile of plastic surgeons working in the state of Goiás. SL: Brazil	Survey data were divided into socioeconomic and demographic profiles. Income, experience, workload were analyzed. Monthly income was compared to the number of weekly surgeries.	Most (59.5%) were under 40 years of age. Income was higher after 10 years of practice. Only 40% use an assistant in at least half of their surgeries.
Purim et al.,^14^ 2016	Profiled recent medical school graduates in southern Brazil and their employment status. SL: Brazil	A 25-item survey of closed questions was applied to 107 recent graduates.	Most (70.1%) entered a medical residency program, of which general surgery (31%) and internal medicine (11%) predominated among male graduates. The main source of income was from practicing medicine (92.5%). Eighty-five percent had ≥ 2 jobs. Both sexes worked more in secondary and tertiary care.
lizuka & Watanabe,^15^ 2016	Analyzed workforce supply with respect to the impact of a new medical residency program. SL: Japan	Using panel data econometrics and fixed effects regressions, the impact of a new medical residency program on physician wages and hospital stays were evaluated in different markets.	During the program, salaries increased due to a shortage of physicians, but decreased soon after. Hospitals in cities where there are fewer medical schools are more affected, and doctors have even given up on remaining active in the city. Regulated prices for hospital services reduce the supply of professionals and worsen patient outcomes.
Falit et al.,^16^ 2016	Analyzed the radiation oncology job market, questioning the expanding number of residency openings vs the clinical capacity of the system. Regulatory concerns regarding payment models and geographic redistribution are addressed. SL: United States	Data from the Surveillance, Epidemiology and End Results program database were used to project the supply of doctors in radiation oncologists between 2015 and 2025.	Demand in this area may be overestimated, and practitioners could receive lower salaries. However, attempts to reduce supply could be subject to antitrust litigation. The equipment sector, the authors point out, would be the winner.
Ramakrishnan et al.,^17^ 2014	Reviewed English language studies on the proportion and status of female physicians in Japan, Scandinavia, Russia/and Eastern Europe. Synthesize evidence on the status of female physicians in regions outside the United States. SL: United States	Review and meta-analysis on the MEDLINE and EBSCO Host Gender Studies Database, from 1992 to 2012, on the participation and experiences of the female medical workforce in countries outside the United States.	The feminization of medicine has brought challenges and changes in medical practice, ranging from how to combine motherhood and the profession to changes in patient care and the lack or excess depending on the medical specialty. The overall proportion of female physicians was lowest in Japan (18%) and highest in Russia (~70%), with women being underrepresented in leadership and prestigious specialties in all three regions.
Macerollo et al.,^18^ 2014	Analyzed interest in and motivation for international mobility among neurology residents and junior neurologists attending the 2011 Spring School for Young Neurologists. SL: European Union	A survey was applied to 118 young neurologists from 34 countries in the European Union, neighboring countries, and the Pan-Arab Union	For most respondents, salary remuneration is not the main motivation for the workforce but depending on the situation in the country of origin, as well as working conditions in the local health care system, access to new technologies, infrastructure, social recognition, among other aspects along these lines.

EBSCO = Elton Bryson Stephens Company; JEL = Journal of Economic Literature; SL = study location ; MEDLINE = Medical Literature Analysis and Retrieval System Online.

**Table 4:** Data synthesis on physician workforce demand in the medical labor market

Article (author, year and JEL codes)	Description, objective and location of the study (SL)	Methodology	Main results
Keralis et al.,^19^ 2018	Described workforce demand for positions in global health programs by analyzing job vacancies. SL: United States	Data collected from online job postings between November 2015 and May 2016.	The number of positions for recent graduates has reduced, with greater demand for technical/area experts, administrators, and project planning positions. Global health training programs are unlikely to equip graduates for the current market.
Fontes et al.,^20^ 2018, I12; I18; O5	Through panel data econometrics, studied the effects of socioeconomic variables on medical care and available resources per municipality (20102016) using the differences-indifferences method. SL: Brazil	Evaluated the impact of the More Doctors Program in municipalities with and without it.	Hospitalizations for conditions sensitive to primary care within the scope of public health care reduced in municipalities with the More Doctors Program. The program also contributed to a broader distribution of doctors nationwide. Specific epidemiological results were not shown, being substituted by the ACSH indicator, a proxy variable for health service coverage, and the degree of resolution of primary health care.
Liu et al.,^21^ 2017	Projected 2030 health workforce demand in 165 countries according to development level. SL: WHO Global Health Observatory	Projection of future health workforce demand for 165 countries from the WHO Global Health Observatory based on projected economic, demographic, and health coverage growth.	In 2030, the global demand will be 80 million workers, but the supply will be 65 million. Growth in the demand and supply of workers is expected to be slower in low-income countries, resulting in inadequate coverage.
Chowdhary et al.,^22^ 2017	Analyzed regional variation in fulltime employment opportunities in radiation oncology (academic and non-academic). SL: United States	Created a database of fulltime radiation oncology job opportunities based on American Society for Radiation Oncology Career Center website postings between March 2016 and March 2017.	Of the 211 posted openings, 38.9%were academic. The highest and lowest overall number were in the South (31.8%) and Northeast (18.5%), respectively, while the highest and lowest of academic jobs were in the South (35.4%) and West (14.6%), respectively. Significant differences in employer type between academic and non-academic jobs.
Warren et al.,^23^ 2015	Discussed how certification changes for radiology residents could affect hiring practices. SL: United States	Opinion article	With the lengthier new board certification process, employers must accept recently graduated radiology residents as merely “board eligible” for up to 18 months. Thus, if the demand for jobs exceeds the supply, lower wages could ensue, and students may accept lower salaries to pay back student loans.
Lucchetti & Lucchetti,^24^ 2014	Studied workforce demand in São Paulo. Evaluated job openings in the city of São Paulo and the characteristics of each opening among medical specialties. SL: Brazil	Used 2011 data from the Medical Job Bank. Cross-sectional descriptive analytical study.	Insufficient supply of internal medicine job openings. General surgery, gynecology/obstetrics and pediatrics specialties are associated with flexible work arrangements, allowing doctors to hold additional jobs. Family medicine and occupational medicine are more associated with to permanent public contracts.
Prabhakar et al.,^25^ 2014	Studied market demand for graduating radiologists to inform workers entering this sector. SL: United States	Data collected from the American College of Radiologists Jobs Board (2010-2013), including the number of new jobs and the total number of views for each posting.	Postings remained stable from 2011 to 2012, as did the number of new applicants registered. On average, the mean number of monthly seekers was approximately double that of new postings in any month. Jobs Board activity significantly increased from 2011 to 2012.
Ali et al.,^26^ 2015	Studied market workforce demand for hepato-pancreatico-biliary surgeons assessed nationwide according to number of procedures. SL: United States	National Inpatient Sample database (2005-2011) queried for cases of pancreas, liver and gallbladder surgery. The cases were mapped to centers offering this treatment.	There is no excess of doctors in the specialized area (HPB) (fellowship training). However, it is desirable that centers where demand is undermet improve multicare training along with surgical practice.

ACSH = hospitalization for ambulatory care sensitive conditions; SL = study location; WHO = World Health Organization.

## DISCUSSION

In the reviewed literature, some authors studied workforce demand (i.e., derived demand from health care system users, which was identified through by job openings or demographic needs), while others studied workforce supply (identified by the number of working physicians, physician job satisfaction, or a sociodemographic characterization of physicians).

Supply side articles covered topics such job openings for physicians, incentives to fill job openings in rural areas, response to government programs or public health policies, and increased or decreased workforce supply in response to increased wages. However, the studies do not explicitly describe remuneration as sufficient or insufficient, but rather the direction of variation. Given that no studies reported income values, probably due to confidentiality issues, some results showed that incentives to retain physicians include job stability and other advantages such as teamwork, the use of cutting-edge technology, proximity to family, and job location.

In the international literature, the terms physician assistant, general practitioner, resident physician, residency, post-residency training, fellowship, oncology radiologist, and specialist appear in different studies. Although the exact meaning of each term may vary from country to country, they coincide in the sense that “general medical practice” moves towards greater specialization and in-depth research.

Despite differences between countries, the concentration of professionals in specific areas is a concern related to workforce supply; i.e., as competition between professionals increases, wages may drop.^11,15^

Barriers-to-entry to new professionals entering the market may include the medical training process, obtaining a diploma, the possibility of being hired as a doctor or a medical practitioner, and board certification examinations.^23^

Labor mobility is individual motivation and ability to migrate to places with more attractive educational and job openings, which can impact the system as a whole by affecting the availability of care providers.^21^ Regarding international mobility, physicians were the second most motivated profession to migrate among European Union countries in 2012. This is relevant since the possibility of obtaining training and specialization in other countries could be aligned with either permanent immigration for professional practice or temporary immigration to for greater specialization.^18^

The “feminization of medicine” is occurring in many countries, including those in the Organisation for Economic Co-operation and Development.^17^ In Brazil, this is corroborated by recent demographic data from the *Conselho Federal de Medicina* (Federal Council of Medicine),^27^ which shows a higher percentage of female than male physicians aged between 27 and 32 years.

Regarding methodology, some authors used applied questionnaires to the workforce or analyzed databases, as in the case of job openings. Data were analyzed using descriptive statistics and regression modeling for linear and non-linear models. Job posting websites were analyzed to collect data on derived demand, which was classified according to medical specialization, employer type (public, private, academic, or non-academic), full-time or part-time status, employment relationship (permanent contract [i.e., according to the Brazilian Consolidation of Labor Laws] or other type) and service type (e.g., outpatient, emergency room, or primary, secondary, or tertiary care). Demand was determined from epidemiological tables, the demographic structure of the study location, and its socioeconomic development level.^21,22^

A shortage of specialized professionals in some regions sometimes arises from “distribution” failures. For example, rural patients must often travel far in search of treatment. The shortage of doctors with a certain specialization in one place and an excess in another also creates an uncomfortable situation.

Changes in public legislation and the norms of professional associations also affect future candidates and the job market, since requirements for additional exams and certifications can impede entry into professional practice. However, such rules become necessary when quality of life is considered paramount.

The impact of wages and forms of employment was less studied. Revenue did not appear to be a priority compared to the opportunity for professional fulfillment, e.g. flexible and inclusive hierarchical structures; suitable means, resources, and instruments for professional practice; teamwork and building career prestige; being close to family and relatives; and greater opportunity for professional training.^18^

The studies themselves were not concerned with aspects of supply or demand as such, but rather elements inherent to these themes, being conducted by physicians in most cases. Aspects of the demand for medical assistance were not identified through the search criteria of this integrative review, although it is of great importance that future studies include epidemiological descriptors (Table 5).

**Table 5:** Analytical synopsis of the 21 reviewed articles

Highlights
Public organizations play an important role in hiring physicians.
Workforce mobility between countries and regions is becoming increasingly relevant.
There is gap in research on diversity in the medical profession.
Studies predicted a shortage of physicians and discussed incentives to increase the number of graduates or, on the contrary, to delay their entry into professional practice, which can reduce wages in case of a surplus or increase them during a natural or artificial shortage (due to barriers, such as new testing protocols, etc.).
The impact of the “feminization of medicine” on care and demographics in different specialties requires further study.
Physicians do not consider salary alone when deciding to accept a position: work conditions, such as infrastructure, location, access to cutting-edge technology, teamwork, and the work environment, are also important.
Management and planning skills are especially important for job openings in international programs.
The World Health Organization has documented a distribution gap in the medical workforce, with a shortage in less developed countries and a surplus in more developed ones.
The case of Brazil
Few studies have characterized physician employment between public and private sectors or investigated the impact of wage stability, increases, or decreases.
Studies show that many physicians have more than one job, but they do not indicate a lack of job openings; the cause or motivation for preferring work in secondary or tertiary care has not been specified.
Public financing is in crisis due to budget deficits and increasing health care costs.
Brazil’s large territory and population (200 million in 2022) pose a challenge to health care system coverage.

## CONCLUSIONS

Studies on the labor market generally focus on employment and unemployment indicators and commonly involve several research axes, such as the labor market and the physician workforce supply, wage models, salaries and fees for service, education, etc. However, considering the inherent complexity of each theme, we chose to update and systematize data through an integrative review on supply and demand for the physician workforce, i.e., in the market in general.

The literature search revealed investigations into physician workforce supply and derived demand. Some studies analyzed job postings, which we considered an aspect of workforce demand. We distinguished between the concepts of derived demand (i.e., as a function of the demand for health care) and demand, clarifying each in the article. We also distinguished between physician workforce supply and demand in the labor market to understand the challenges of the profession without suggesting a marketing vision for health care, thus contributing to research on medical training and practice in Brazil.^28^ Most of the reviewed studies on medical specialties and job supply were from outside Brazil. The review consisted of studies published prior to the COVID-19 pandemic (2014 to 2018) to avoid its effects. New Brazilian studies in this line of research are needed to fill knowledge gaps regarding fees for procedures, employment types, physician quality of life, and public health policy issues.

As in most professions, physicians are faced with the dilemma that wages tend to decrease as the number of professionals in the area increases, with all other things being equal (innovations, differentiating attributes, etc.). In the health sector, market interventions to increase the general well-being of the population can also occur, since free markets cannot resolve health care access for the most vulnerable populations – not only socioeconomic vulnerability, but interventions to ensure professional dignity. New studies may investigate the profession in the context of the price and cost of innovation, procedures, competition, and dignified work conditions.

Future Brazilian studies will require ethics committee approval and the collaboration of actors in the health system for qualitative and quantitative research, in addition to medical demographics. New multidisciplinary research proposals in medical economics would be ideal, including support and broader understanding by academic journals.

## References

[1] Pindyck RS, Rubinfeld DL (2005). Microeconomia.

[2] Folland S, Goodman AC, Stano M (2008). A economia da saúde.

[3] Hubbard RG, O’Brien AP (2010). Introdução à economia.

[4] Botelho LLR, Cunha C, Macedo M (2011). O método da revisão integrativa nos estudos
organizacionais. Gest Soc.

[5] Hunt EK (1981). História do pensamento econômico.

[6] Leung J, Kariyasawan S, Forstner D, Chee R, James M (2018). Employment for radiation oncologists in Australia and New Zealand: recent
graduates survey of experiences and perspectives. J Med Imagin Gradiat Oncol.

[7] Chen A, Lo Sasso AT, Richards MR (2018). Supply-side effects from public insurance expansions: evidence from
physician labor markets. Health Econ.

[8] Kalb G, Kuehnle D, Scott A, Cheng TC, Jeon SH (2018). What factors affect physicians’ labour supply: comparing structural
discrete choice and reduced-form approaches. Health Econ.

[9] L’hopital C (2017). Dónde, cómo y por qué trabajan los médicos
generalistas formados por el Estado. Archiv Med Fam Gen.

[10] Suciu ŞM, Popescu CA, Ciumageanu MD, Buzoianu AD (2017). Physician migration at its roots: a study on the emigration preferences and
plans among medical students in Romania. Hum Resour Health.

[11] Maceira D, Palácios A, Urrutia M, Espínola N, Nievas M (2017). Descentralización y estructura de lãs remuneraciones
médicas en Argentina: um análisis comparado en cinco
jurisdicciones. Rev Argent Salud Publica.

[12] Higgins MCS, Hwang WT, Richard C, Chapman CH, Laporte A, Both S (2016). Underrepresentation of women and minorities in the United States IR
academic physician workforce. J Vasc Interv Radiol.

[13] Arruda FCF, Paula PR, Porto CC (2016). Perfil do cirurgião plástico no estado de Goiás,
Brasil. Rev Bras Cir Plast.

[14] Purim KSM, Borges LMC, Possebom AC (2016). Perfil do médico recém-formado no sul do Brasil e sua
inserção profissional. Rev Col Bras Cir.

[15] Iizuka T, Watanabe Y (2016). The impact of physician supply on the health care system: evidence from
Japan’s New Residency Program. Health Econ.

[16] Falit BP, Pan HY, Smith BD, Alexander BM, Zietman AL (2016). The radiation oncology job market: the economics and policy of work force
regulation. Int J Radiation Col Biol Phys.

[17] Ramakrishnan A, Sambuco D, Jagsi R (2014). Women’s participation in the medical profession: insights from experiences
in Japan, Scandinavia, Russia, and Eastern Europe. J Womens Health (Larchmt).

[18] Macerollo A, Varga ET, Struhal W, Györf O, Kobeleva X, Sellner J (2014). International Issues: cross-border mobility of junior neurologists with in
and to the European Union. Neurology.

[19] Keralis JM, Riggin-Pathak BL, Majeski T, Pathak BA, Foggia J, Cullinen KM (2018). Mapping the global health employment market: an analysis of global health
jobs. BMC Public Health.

[20] Fontes LFC, Conceição OC, Jacinto PA (2018). Evaluating the impact of physicians’ provision on primary healthcare:
evidence from Brazil’s More Doctors Program. Health Econ.

[21] Liu JX, Goryakin Y, Maeda A, Bruckner T, Scheffler R (2017). Global Health workforce labor market projections for 2030. Hum Resour Health.

[22] Chowdhary M, Chhabra AM, Switchenko JM, Jhaveri J, Sen N, Patel PR (2017). Domestic job shortage or job maldistribution? A geographic analysis of the
current radiation oncology job market. Int J Rad Oncol Biol Phys.

[23] Warren LA, Patel TY, Layman MS, Patel MY (2015). The job market of the future: how will the recent change in boards
influence the job market for new graduates?. J Am Coll Radiol.

[24] Lucchetti ALG, Lucchetti G (2014). Mercado de trabalho médico no estado de São Paulo:
análise das ofertas de empregos contidas no site “Banco de Empregos
Médicos”. Rev Soc Bras Clin Med.

[25] Prabhakar AM, Oklu R, Harvey HB, Harisinghani MG, Rosman DA (2014). The radiology job market: analysis of the ACR jobs board. J Am Coll Radiol.

[26] Ali N, O’Rourke C, El-Hayek K, Chalikonda S, Jeyarajah DR, Walsh RM (2015). Estimating the need for hepato-pancreatico-biliary surgeons in the
USA. HPB (Oxford).

[27] Scheffer M (2020). Demografia médica no Brasil.

[28] Zyngier SP, Scheffer MC, Tagusagawa LK, Zhang JMF, Cassenote AJF, Matijasevich A (2021). Perfil dos médicos formados na FMUSP e ingresso na residência
médica. Rev Med (São Paulo).

